# Conceptualization, Operationalization, and Utilization of Race and Ethnicity in Major Epidemiology Journals, 1995–2018: A Systematic Review

**DOI:** 10.1093/aje/kwac146

**Published:** 2022-08-08

**Authors:** Rae Anne M Martinez, Nafeesa Andrabi, Andrea N Goodwin, Rachel E Wilbur, Natalie R Smith, Paul N Zivich

**Keywords:** ethnicity, health equity, race, scientific communication, systematic reviews

## Abstract

Despite repeated calls by scholars to critically engage with the concepts of race and ethnicity in US epidemiologic research, the incorporation of these social constructs in scholarship may be suboptimal. This study characterizes the conceptualization, operationalization, and utilization of race and ethnicity in US research published in leading journals whose publications shape discourse and norms around race, ethnicity, and health within the field of epidemiology. We systematically reviewed randomly selected articles from prominent epidemiology journals across 5 periods: 1995–1999, 2000–2004, 2005–2009, 2010–2014, and 2015–2018. All original human-subjects research conducted in the United States was eligible for review. Information on definitions, measurement, coding, and use in analysis was extracted. We reviewed 1,050 articles, including 414 (39%) in our analyses. Four studies explicitly defined race and/or ethnicity. Authors rarely made clear delineations between race and ethnicity, often adopting an ethnoracial construct. In the majority of studies across time periods, authors did not state how race and/or ethnicity was measured. Top coding schemes included “Black, White” (race), “Hispanic, non-Hispanic” (ethnicity), and “Black, White, Hispanic” (ethnoracial). Most often, race and ethnicity were deemed “not of interest” in analyses (e.g., control variables). Broadly, disciplinary practices have remained largely the same between 1995 and 2018 and are in need of improvement.

## Abbreviations

COVID-19: coronavirus disease 2019
OMB: Office of Management and Budget

***Editor’s*** ***note:** An invited commentary on this article and the authors' response will appear in an upcoming issue.*

After decades of research, it is clear that race and ethnicity are salient constructs in understanding current systems of social stratification and health (1–3). The consequences of racial and ethnic stratification are most obvious across a wide range of health outcomes and time in health disparities research (4–10). Currently, the tragic ramifications of racial and ethnic health stratification are being illuminated by the ongoing coronavirus disease 2019 (COVID-19) pandemic (11–14).

Despite evidence of race and ethnicity’s salience to health scholarship, these constructs appear undermotivated in their use (15). Epidemiologists have debated the usefulness of collecting, analyzing, and interpreting data on race and ethnicity over the last few decades (16–35). Some scholars have called for abandoning racial and ethnic data, arguing that such categories perpetuate racism, simply capture the consequences of socioeconomic status, or are better reflected by measuring genetic ancestry (16, 17, 24, 29, 36). However, such calls have been repeatedly met with fierce rebuttal; the social constructs of race and ethnicity are vital in addressing racial and ethnic health disparities (3, 18, 25, 37). Furthermore, they are necessary to understand how racism influences scientific practices (e.g., systematic exclusion of some populations from the production of knowledge (38, 39), the development of methodology (40–42), and unethical medical experimentation or treatment (43–45)).

Underlying these debates is the recognition that when race and ethnicity data are incorporated into epidemiologic research, it is simply not done well (16, 46–48). LaVeist (49) challenged population health researchers to “do a better job” of conceptualizing race, understanding the nuances of racial and ethnic measurements, and interpreting findings with care. Such guidelines and recommendations continue to arise (15, 16, 25, 29, 35, 37, 46–48, 50–61); select recommendations are summarized in Table 1.

**Table 1:** Prior Recommendations and Guidance for the Use of Race and Ethnicity in Epidemiologic Research, 1990–2021^a^

**Recommendation**	**References**
Conceptualization	
Provide a definition of race.	15, 16, 25, 37, 47, 48, 50–52
Acknowledge that race is a social construct.	29, 51, 53–55
Provide a definition of ethnicity.	16, 25, 35, 50, 52
Race and ethnicity should be acknowledged as distinct social constructs and should not be used as synonyms.	50, 55–57
Operationalization	
Collect quality data on race and ethnicity.	38, 54, 58, 59, 114, 115
Describe how race is measured.	29, 37, 46, 47, 50, 55, 60, 116
Describe how ethnicity is measured.	55–57, 116
Analyses and interpretation	
Consider the appropriateness of controlling for race in the analysis.	29, 33, 48, 117
Explore between-group differences and within-group heterogeneity whenever possible.	48, 59
Provide interpretations of race-associated differences (even when race is a control variable).	29, 46, 48, 60
Justification	
Justify including race as a variable and define the variable in the context of the study.	15, 29, 47, 52, 56, 60, 116
Justify excluding respondents from the study design or analysis on race and/or ethnicity.	38, 48, 114, 115
Acknowledge the limitations of racial and/or ethnic measurement.	55–57
Contextual considerations	
Collect quality data on and examine other social determinants of health and structural factors that are often associated with race. This includes but is not limited to socioeconomic status, discrimination, racism, culture, neighborhood/place, political factors, nativity, and acculturation.	15, 25, 29, 37, 47, 48, 50, 51, 53–55, 58, 60, 61
Acknowledge the possible influence of personal values and biases on scientific research and policy-making.	56, 57, 61

^a^ This is not a comprehensive list of all recommendations for epidemiology or public health research published between 1990 and 2021. Furthermore, this is not even a full listing of all recommendations within the cited articles; many of the cited articles have additional, more nuanced guidance. This table is simply meant to demonstrate patterns. Similar guidance exists in other disciplines, including but not limited to medicine (110, 118, 119), nutrition (120), and psychology (121).

This paper responds to these calls to “do a better job” with empirical evidence characterizing the state of conceptualization, operationalization, and utilization of race and ethnicity since LaVeist’s 1996 review (49). Previous studies on this topic have had several key limitations (46, 47, 55, 62–67). First, the majority of these works review literature published within a single journal, which falls short of assessing trends that are prolific throughout a specific discipline (47, 62, 63, 65). Second, works that attempt to review literature from 2 or more journals are limited in their temporal scope (e.g., 4–5 years) (55, 64, 66).

In this paper, we systematically describe the conceptualization, operationalization, and utilization of race and ethnicity over the past 25 years in 5 leading general epidemiology journals that shape discourse around race, ethnicity, and health within the field. We asked the following questions about race and ethnicity in epidemiology over time: 1) What proportion of epidemiologic research studies include data on race and ethnicity? 2) What proportion of studies provide a conceptualization (i.e., definition) of race and ethnicity? 3) How are race and ethnicity data operationalized (i.e., measured and coded)? 4) How are race and ethnicity data utilized in analyses?

## METHODS

### Conceptualization of race and ethnicity

Race is a relational, time-varying, multidimensional social construct, predicated upon assigning social meaning to an arbitrary phenotype or set of phenotypes (48, 68, 69). The social meaning is contextually specific to the time period and social-cultural-political context. The boundaries of racial groups are enforced through social and structural interactions to maintain the privilege, power, and resource aggregation of the dominant group (3, 70). Similarly, ethnicity is a relational, time-varying, multidimensional social construct, rooted in a sense of belonging around elements of shared culture (e.g., language, religion, dress, values, or beliefs) and of place (69, 71, 72). Neither race nor ethnicity is determined by biology.

We conceptualize “Hispanic” and “Latino/a/x” to be panethnic identities, not racial identities, in line with the US Office of Management and Budget (OMB) (73–75). As such, individuals who identify as Hispanic or Latino/a/x can be of any racial identity. For example, someone who identifies as “White and Cuban,” “Black and Panamanian,” or “Tohono O’odham (Indigenous) and Mexican” could all fall under the umbrella of Hispanic or Latino/a/x. Despite the terms’ frequently being considered synonymous, “Hispanic” refers to people who are Spanish-speaking and/or descended from Spain (i.e., it includes Spain but excludes Brazil and Portugal), while “Latino/a/x” emphasizes geography over language and colonialist history by referring to people who are from or descended from Latin America (i.e., it includes Brazil but excludes Spain and Portugal) (76). We further view “African American” as a US-centered ethnic identity that reflects the shared history of forced removal and enslavement in the United States or of an acculturated “American” experience. We define “Black” as a racial identity based on the perceptions of shared phenotype (77, 78). Recent immigrants or permanent residents from the Caribbean, Brazil, or Nigeria may racially identify as or be racialized by others in the United States as “Black” but may not ethnically identify as “African American.”

### Study design

**Figure 1:**
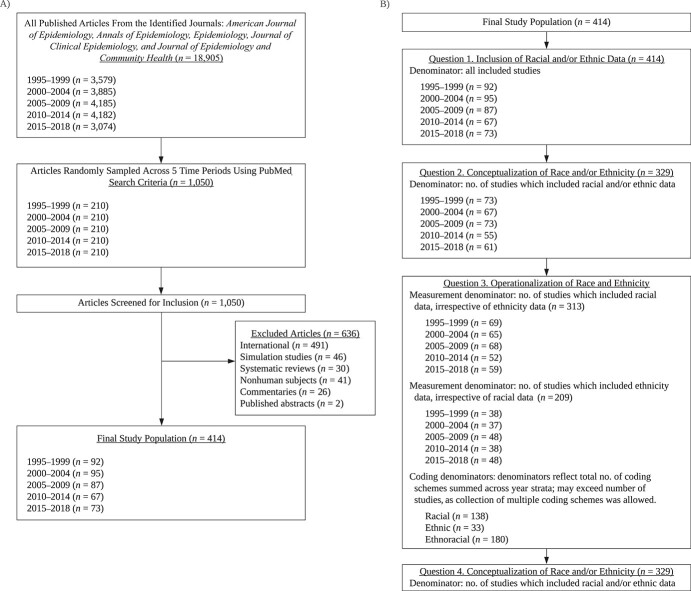
Outline and conduct of a study on the use of race and ethnicity in research published in 5 leading general epidemiology journals, 1995–2018. A) Study sampling and inclusion strategy. The total population of articles included all articles published in the 5 identified journals between January 1, 1995, and December 31, 2018. In total, 18,905 articles were identified; this included articles that did not meet study eligibility criteria (i.e., US-based, original human subjects research). B) Denominators used in the study, by research question. For question 3, coding schemes were grouped on the basis of question 1 results (i.e., race, ethnicity, or ethnoracial data). Studies with racial coding schemes included racial data and may have included ethnicity data, but did not combine the two into an ethnoracial construct. Similarly, studies with ethnic coding schemes included ethnic data and may have included racial data, but kept them as distinct constructs. Studies denoted as “ethnoracial” combined or conflated racial and ethnic data. Denominators for question 4 were identical to those for question 2 and are therefore not shown.

We systematically reviewed a sample of US human-subjects research published in 5 prominent epidemiology journals: the *American Journal of Epidemiology*, *Annals of Epidemiology*, *Epidemiology*, the *Journal of Clinical Epidemiology*, and the *Journal of Epidemiology and Community Health*. Journals were selected on the basis of their impact factor and reputation, an approach consistent with systematic reviews on disciplinary norms surrounding null hypothesis significance testing (79, 80). Rather than reviewing all articles published in these journals, we selected a stratified random sample across the following 5-year increments: 1995–1999, 2000–2004, 2005–2009, 2010–2014, and 2015–2018. From across the selected journals, 210 articles were sampled for each time stratum via PubMed (National Library of Medicine, Bethesda, Maryland) (Figure 1A). Full search details are provided in Web Appendix 1, available at https://doi.org/10.1093/aje/kwac146.

Of sampled articles, only US original human-subjects research was eligible for review (excluded: commentaries, systematic reviews, meta-analyses, and simulation studies). The contextually dependent nature of race and ethnicity informed our decision to restrict the data to US-based studies. Exclusion criteria aligned with prior literature (46, 47, 62–67).

Of 1,050 randomly sampled articles, 414 (39%) were included. Excluded articles were primarily non-US research (Figure 1A).

### Data abstraction process

Prior to data collection, all reviewers practiced on 5–10 articles to ensure consistency. Each article was reviewed twice; supplementary materials were not reviewed. The first reviewer extracted article information into a standardized, electronic REDCap (Vanderbilt University, Nashville, Tennessee) form (81, 82) (Web Appendix 2) using an abstraction protocol developed by the authors. Secondary reviewers read the same article and reviewed REDCap entries to ensure data quality. Reviewers were rotated every 75 articles. If a discrepancy in the data entry was identified (e.g., a typographical error or misclassification) by the second reviewer, the records were discussed by the pair. If the discrepancy was not resolved, the record was discussed by all authors. Group decisions for standardized entry of special cases were recorded in the abstraction protocol for future reference. A third data quality check was performed prior to completion of all records by the first author.

### Data extraction

Data collection was completed on the basis of the information present in the final, published manuscript. Reviewers were instructed to not rely on prior knowledge of particular data sets for the collection of measurement data.

#### Article characteristics.

Basic article information (title, first author, publication year, PubMed identification number (PMID), journal) was collected using PubMed. Additional information on study design (“cohort,” “randomized controlled trial (RCT),” “ecological,” “case-control”), data source, sample size, type of health outcome (classified as “health behavior,” “mental or physical health,” “health-care access or utilization,” or “other”), and specific health outcome (free response) was collected.

#### Question 1: inclusion of racial and ethnic data.

For each article, reviewers were asked 3 yes/no questions: “Did they measure RACE?”; “Did they measure ETHNICITY?”; and “Did they combine RACE and ETHNICITY?”. If data on participants’ race was included in any capacity (e.g., in text or tables), reviewers were instructed to mark “yes.” Parallel instructions were given for ethnicity. If both racial and ethnic data were included, reviewers were instructed to examine whether racial and ethnic data or language was combined or conflated.

#### Question 2: conceptualization of race and ethnicity.

For each article, reviewers were asked, “Did the authors provide a working definition of race?” (yes/no). Reviewers were instructed to look for explicit definitions of race or statements reflecting the authors’ perspective (e.g., “Race is a social construct…” or “...biological traits such as race…”). If yes, the verbatim definitions were recorded. Instructions for ethnicity were the same.

#### Question 3: operationalization.

Our assessment of operationalization first examined the measurement of race and ethnicity. We employed Roth’s framework (68) to collect information on racial measurements used in the sampled studies. Roth broadly postulates that race is a multidimensional, social construct that can be broken down into numerous measures, each of which captures unique information about an individual’s complex racial identity (13). Roth enumerates 6 measures of race as described in Table 2. Three additional measures were considered in data collection: “not stated/unclear,” “not used,” and “unclear between identity and self-classification.” We further adapted Roth’s framework to ethnicity, which similarly resulted in 9 measures (Table 2). If a study used multiple measures of race (or ethnicity), all were collected.

**Table 2:** Definitions of Measures of Race and Ethnicity in Epidemiologic Research

**Measure**	**Definition**
Measures of race^a^	
Identity	Subjective self-identification assessed through an open-ended question (i.e., free response)
Self-classification	Self-identification assessed through a closed-ended question
Observed	Race identified by a third party (e.g., interviewer) based on appearance alone or through interaction
Phenotype	Skin tone or other physical characteristics (e.g., hair texture, bone structure) assessed alone or in combination
Reflected	The race you believe others assume you to be; respondent’s understanding of how they are view by others
Ancestry	As informed by familial history, genetic testing, or blood quantum
Unclear/not stated^b^	Insufficient information or no information on how race was measured
Identity versus self-classification^b^	Race was self-identified, but unclear whether the question was open- or closed-ended
Measures of ethnicity^c^	
Identity	Subjective self-identification assessed through an open-ended question (i.e., free response)
Self-classification	Self-identification assessed through a closed-ended question
Country of origin	The country from which a person originally comes or “nationality”
Observed	Ethnicity identified by a third party (e.g., interviewer) based on appearance alone or through interaction
Reflected	The ethnicity you believe others assume you to be; respondent’s understanding of how they are view by others
Ancestry	As informed by familial history
Unclear/not stated^b^	Insufficient information or no information on how ethnicity was measured
Identity versus self-classification^b^	Ethnicity was self-identified, but unclear whether the question was open- or closed-ended

^a^ Unless otherwise noted, measures originated from Roth (68).

^b^ These measures were created by the authors for data collection purposes.

^c^ Ethnicity measures were adapted from Roth (68); other measures of ethnicity may exist.

Second, our assessment of operationalization examined the verbatim coding schemes of race and ethnicity. Data were collected from articles into free response text boxes. If, under question 1, an article was marked as combining race and ethnicity into a single ethnoracial construct, the coding scheme entered for race and ethnicity into the separate text boxes was identical. Information on capitalization was not collected. No attempt was made to collapse coding schemes on the basis of similarity.

In some articles, coding schemes for race and/or ethnicity were not consistent throughout the article. At times, coding schemes differed between the authors’ table presenting participants’ demographic characteristics (usually Table 1) and their analyses; in other cases, different coding schemes were used for different analyses within the same article. If multiple coding schemes were used in analyses, all were recorded. If a variable was only used as a descriptor (i.e., not in analyses), then the coding scheme from the demographics table was collected. If the coding scheme differed between the demographics table and analyses, only the analytical coding scheme was collected.

#### Question 4: use in analyses.

The role of race and ethnicity in each study’s analyses was classified into one of 4 categories: “of interest,” “not of interest,” “exclusion,” and “other.” “Of interest” was selected when race and/or ethnicity was used by the study authors as a focal variable (e.g., group comparisons, effect measure modification, mediation, instrumental variable). “Not of interest” was selected when race and/or ethnicity was used by the study authors as a matching criterion, a confounder, or simply a descriptive covariate. When race and/or ethnicity was used to exclude participants from a study (primary data collection) or analyses (secondary data), “exclusion” was selected. If race was included in a regression model, the reference category of the racial, ethnic, or ethnoracial variable was recorded.

### Software

Cleaning of open-ended free response text and sampling was conducted in Python 3.5.2 (83) (Python Software Foundation, Beaverton, Oregon) using the Biopython (84) and NumPy (85) libraries. Analyses were performed in R, version 4.0.2 (86) (R Foundation for Statistical Computing, Vienna, Austria), with the packages ggwordcloud (87), tableone (88), tidytext (89), and tidyverse (90).

## RESULTS

Characteristics of articles are presented in Table 3. Across time periods, the majority of articles were cohort studies (range, 72%–93%) and examined a physical or mental health outcome (range, 64%–79%). Ninety-three studies used primary data or did not list a specific secondary data source. Of the 321 articles which named secondary data sources, the most prevalent were the Nurses’ Health Study (*n* = 13), the National Health and Nutrition Examination Survey (*n* = 11), and the Atherosclerosis Risk in Communities Study (*n* = 11) (data not shown).

**Table 3:** Study Design Characteristics of Articles Included in an Analysis of Race and Ethnicity in Epidemiologic Research (*n* = 414), 1995–2018

	**Time Period**									
	**1995–1999** **(*n* = 92)**		**2000–2004** **(*n* = 95)**		**2005–2009** **(*n* = 87)**		**2010–2014** **(*n* = 67)**		**2015–2018** **(*n* = 73)**	
**Study** **Characteristic**	**No. of** **Studies**	**%**	**No. of** **Studies**	**%**	**No. of** **Studies**	**%**	**No. of** **Studies**	**%**	**No. of** **Studies**	**%**
Study design										
RCT	3	3	1	1	1	1	3	4	0	0
Cohort study	66	72	73	77	68	78	56	84	68	93
Case-control study	22	24	20	21	18	21	8	12	5	7
Ecological study	1	1	1	1	0	0	0	0	0	0
Study outcome^a^										
Health behavior	4	4	11	12	8	9	4	6	7	10
Physical or mental	71	77	74	78	69	79	53	79	47	64
Health-care access	4	4	2	2	5	6	3	4	4	5
Other	14	15	13	14	8	9	10	15	19	26
Sample size, no. of persons^b^										
<1,000	38	41	38	40	17	20	17	25	19	26
1,000–5,000	33	36	32	34	31	36	16	24	20	27
5,001–10,000	8	9	11	12	7	8	9	13	8	11
10,001–100,000	18	20	20	21	23	26	24	36	20	27
>100,000	4	4	5	5	10	11	10	15	13	18
Missing data	2	2	1	1	1	1	2	3	2	3

Abbreviation: RCT, randomized controlled trial.

^a^ Study outcomes were classified as health behaviors (e.g., smoking, dietary intake, physical activity, sexual behaviors), mental or physical health (e.g., obesity, high blood pressure, cancer, depression), health-care access or utilization (e.g., health insurance status, number of primary care visits, quality of care), or other. Study outcomes are not mutually exclusive, and percentages may sum to more than 100.

^b^ Some studies listed more than 1 analytical sample size; percentages may sum to more than 100.

###  

#### Question 1: inclusion of racial and ethnic data.

**Figure 2:**
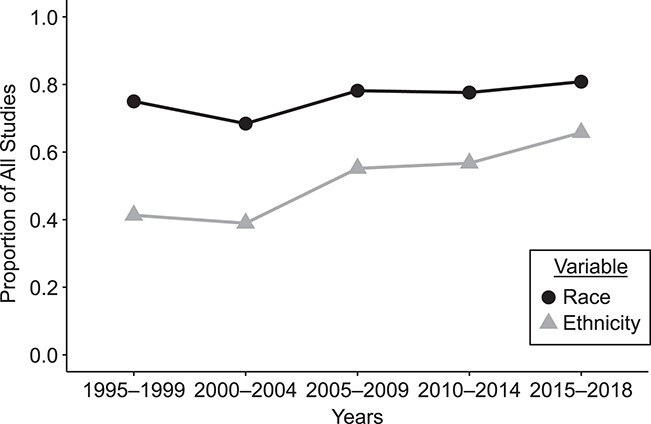
Proportion of epidemiologic studies that included information on the study population’s race and/or ethnicity over time, 1995–2018. Across all strata, 414 articles met the inclusion criteria. Of those, 313 included at least racial data (irrespective of including ethnicity data) and 209 included at least ethnicity data (irrespective of racial data).

The proportion of epidemiologic studies that included participants’ racial data remained relatively stable between 1995 and 2018 (range, 68%–81%; Figure 2). At least 19% of articles in every time period did not include racial data. These articles were more likely to study “other” health outcomes (range, 16%–29%) but otherwise did not differ from the overall sample (Web Table 1). In the same time frame, the proportion of studies that included information about participants’ ethnicity increased (range, 39%–66%; Figure 2).

Studies that included both racial and ethnic data typically combined race and ethnicity into a single ethnoracial construct (range, 83%–96%). Only 17 studies (4.1%) across all strata measured both race and ethnicity and kept them as separate constructs.

#### Question 2: conceptualization of race and ethnicity.

Across all 329 studies which included data on participants’ race and/or ethnicity, only 4 studies defined race and/or ethnicity. In one case, Johnson et al. combined race and ethnicity into an ethnoracial construct and provided a brief definition: “Using race/ethnicity as a proxy measure of respondent culture…” (91, p. 661). Two studies defined ethnicity using explicit reference to the US OMB’s definitions (92, 93). In the final study, Kandula et al. noted that “ethnicity is used as a marker of cultural beliefs about health” (94, p. 192). The remaining 325 studies included race and/or ethnicity data without providing construct definitions.

#### Question 3: operationalization.

Measurement of race was unclear or not stated in the vast majority of studies (range, 76%–81%; Table 4). Racial measurement was also commonly unclear between identity and self-classification (range, 13%–22%). For example, in studies that indicated race was “self-reported,” it was frequently ambiguous whether the measure was “open” (i.e., an open-ended free response identity) or “closed” (i.e., selection from preset categories). In a few studies, the measures of “self-classification” and “ancestry” were observed.

**Table 4:** Measures of Race and Ethnicity Used in Epidemiologic Research Studies (*n* = 414), 1995–2018

	**Time Period**									
	**1995–1999** **(*n* = 92)**		**2000–2004** **(*n* = 95)**		**2005–2009** **(*n* = 87)**		**2010–2014** **(*n* = 67)**		**2015–2018** **(*n* = 73)**	
**Race or Ethnicity** **Measure** ^**a**^	**No. of** **Studies**	**%**	**No. of** **Studies**	**%**	**No. of** **Studies**	**%**	**No. of** **Studies**	**%**	**No. of** **Studies**	**%**
Race	69	100	65	100	68	100	52	100	59	100
Identity	0	0	0	0	0	0	0	0	0	0
Self-classification	4	6	2	3	2	3	0	0	0	0
Observed	0	0	1	2	0	0	0	0	0	0
Phenotype	0	0	0	0	0	0	0	0	0	0
Reflected	0	0	0	0	0	0	0	0	0	0
Ancestry	1	1	0	0	0	0	0	0	0	0
Unclear/not stated	56	81	51	78	52	76	41	79	46	78
Identity versus self-classification^b^	9	13	12	18	15	22	11	21	13	22
Ethnicity	38	100	37	100	48	100	38	100	48	100
Identity	0	0	0	0	0	0	0	0	0	0
Self-classification	3	8	3	8	2	4	0	0	0	0
Country of origin	2	5	0	0	1	2	1	3	1	2
Observed	0	0	1	3	0	0	0	0	0	0
Reflected	0	0	0	0	0	0	0	0	0	0
Ancestry	2	5	1	3	0	0	0	0	1	2
Unclear/not stated	26	68	28	76	34	71	30	79	38	79
Identity versus self-classification^b^	7	18	5	14	13	27	9	24	10	21

^a^ Selection of multiple measures was allowed; percentages may sum to more than 100.

^b^ For racial identity versus self-classification, race was noted as self-identified by a participant, but it was unclear whether the question was open- or closed-ended. The same applies to ethnic identity versus self-classification.

Findings for ethnicity were similar (Table 4). Across time, the measurement of ethnicity was unclear or not stated in the majority of studies (range, 68%–79%), followed by “unclear between identity and self-classification” (14%–27%). A few studies used “country of origin,” “ancestry,” or “self-classification” to measure ethnicity.

The coding schemes of sampled articles were collapsed across strata and grouped on the basis of the adoption of a racial, ethnic, or ethnoracial framing (i.e., question 1 results). The most frequently observed coding schemes are summarized in Table 5. For racial and ethnic codings, the coding schemes determined to be “most frequent” were those representing more than 5% of all captured coding schemes (not studies). Given the high degree of heterogeneity in the ethnoracial coding schemes, “most frequent” were those representing more than 2% of all captured coding schemes. All racial and ethnoracial coding schemes are additionally listed in Web Tables 2 and 3.

**Table 5:** The Most Common Racial, Ethnic, and Ethnoracial Coding Schemes Used in Epidemiologic Research Studies (*n* = 414), 1995–2018^a^

**Coding Scheme**	**No. of Coding Schemes**	**%**
Racial coding schemes^b^		
Black, White	29	21
Black, other, White	18	13
Not stated	17	12
White	16	12
Nonwhite, White	15	11
Ethnic coding schemes^c^		
Mexican American	5	15
Hispanic, non-Hispanic	4	12
Not stated	4	12
Ethnoracial coding schemes^d^		
African American, White	10	6
African American, other, White	7	4
Asian, Black, Hispanic, other, White	7	4
Black, Hispanic, White	7	4
Black, Hispanic, other, White	6	3
Hispanic, non-Hispanic Black, non-Hispanic White, other	6	3
African American	5	3

^a^ Information on capitalization was not collected. No attempt was made to collapse coding schemes based on similarity.

^b^ There were 33 unique racial coding schemes identified from among 138 individual coding schemes belonging to 137 studies. These studies included racial data and may have included ethnicity data, but did not combine the two into an ethnoracial construct.

^c^ There were 18 unique ethnic coding schemes found from among 33 individual coding schemes belonging to 33 studies. These studies included ethnic data and may have included racial data, but did not combine the two.

^d^ There were 129 unique ethnoracial coding schemes identified from among 180 total coding schemes belonging to 176 studies.

Across all strata, 33 unique racial coding schemes were identified from among 138 individual schemes belonging to 137 studies. The most commonly observed was “Black, White” (*n* = 29), “Black, other, White” (*n* = 18), “NS” (not stated; *n* = 17), “White” (*n* = 16), and “nonwhite, White” (*n* = 15) (Table 5). Articles typically arrived at a coding scheme of solely “White” in one of 2 ways. Either the study was restricted to only White participants or authors did not provide sufficient description. As in the study by Ottman et al., a common practice was to state “eighty-seven percent of the probands were white” (95, p. 236) without describing the racial composition of the rest of the sample.

The most frequently observed ethnic coding was “MexicanAmerican” (*n* = 5), “Hispanic, non-Hispanic” (*n* = 4) and “NS: not stated” (*n* = 4) out of 18 unique ethnic coding schemes (Table 5). Similar coding schemes included “Hispanic” (*n* = 3), “non-Hispanic” (*n* = 2), “Hispanic/Latino” (*n* = 1), and “Latino” (*n* = 1). Much in the same way as US racial discussions are rooted in the “Black, White” binary, US discussions of ethnicity appear to be rooted in a “Hispanic, non-Hispanic” binary.

The ethnoracial coding schemes had a high degree of heterogeneity, with 129 unique racial coding schemes identified from among 180 individual schemes belonging to 176 studies. Many of the most common were of a similar variation: “Asian, Black, Hispanic, other, White” (*n* = 7); “Black, Hispanic, White” (*n* = 7); “Hispanic, non-Hispanic Black, non-Hispanic White, other” (*n* = 6); and “Black, Hispanic, other, White” (*n* = 6; Table 5). In these coding schemes, “Hispanic” (a panethnic group) is compared with “White” and “Black” (racial groups) and all other individuals are grouped into an ambiguous “other” category.

A fair number of racial and ethnoracial coding schemes used the term “Caucasian” (8 unique racial coding schemes and 17 unique ethnoracial codings; Web Tables 2 and 3). Additionally, a few racial and ethnoracial studies used “European” (1 racial coding scheme and 2 ethnoracial coding schemes). We interpreted the use of “Caucasian” and “European American” to be signifiers of race, specifically as a synonym for “White” (33). Across racial and ethnoracial coding schemes, the reference group was predominantly White (including “White,” “non-Hispanic White,” and “Caucasian;” *n* = 64).

As a part of this analysis, it is important to acknowledge those we did not observe mentioned in any of the sampled articles. This includes Black Latino/a/x, Indigenous Latino/a/x, and Middle Eastern and North African individuals. We also rarely found ethnic heterogeneity discussed for Asian or Indigenous individuals. We find this particularly striking, because our sample is a representative sample of US research over an approximately 25-year period in some of the most prominent journals of this discipline. This may signal that the health and well-being of certain US populations is not being elevated to national discussion and/or is dramatically understudied.

#### Question 4: use in analyses.

Most often race and ethnicity were not of primary interest (i.e., confounder, covariate, matching criteria) in analyses (Web Table 4). Of the 40 studies across strata which used race and/or ethnicity as an exclusion criterion, one-fourth were restricted to a solely White population (*n* = 11). This practice appeared to decline over time (1995–1999, *n* = 5; 2000–2004, *n* = 4; 2005–2009 and 2010–2014, *n* = 1). Other studies used race and/or ethnicity as an exclusion criterion in order to take a deeper dive into the health of specific communities. Studies of this nature focused on the health of “Black women,” “Black/African Americans,” “Japanese Americans,” “Mexican Americans,” “Navajo,” “Oahu residents of Japanese or Okinawan ancestry,” “Puerto Ricans,” and “American Indian” individuals. The remaining 10 studies restricted the study sample to 2 or more groups for specific comparisons.

## DISCUSSION

Despite recurring calls to “do a better job” and various recommendations for action (Table 1), US epidemiologic research published in prominent journals throughout the past 25 years has remained largely unchanged. Inclusion of racial and ethnic data increased during the period 1995–2018, but authors typically did not provide definitions and largely did not describe how race and/or ethnicity was measured (e.g., explicitly mentioning whether open- or closed-ended self-report questions were used). Furthermore, racial coding schemes appeared to be centered on Whiteness through codings like “White, nonwhite” and the use of “White” as the common referent. Similarly, we saw common usage of an ambiguous “other” category, with authors largely failing to describe or justify their coding decisions. This may point to problematic underlying practices that center White lives and experiences over others, despite the rapidly changing racial and ethnic landscape of the United States (96).

In this review, we observed that while the proportion of studies which include both race and ethnicity is increasing, only 17 studies across strata did not collapse race and ethnicity into an ethnoracial construct. The vast majority of sampled articles also did not define race and/or ethnicity. This is concerning, given the theoretical and tangible differences between them. Race, ethnicity, and ethnorace as distinct theoretical constructs have different embedded assumptions. Defining and treating race and ethnicity as separate constructs assumes that they capture unique information that relates to health-associated exposures, outcomes, and mechanisms in different ways. The notion of ethnorace purports that ethnic characteristics (i.e., language, religion) and racial characteristics (i.e., skin tone, bone structure) inform the perception of one another and cannot be separated (97). This framing assumes that race and ethnicity are capturing intertwined information or the same information and have identical relationships to health.

Compounding this issue, authors in the sampled articles also generally did not justify their choices with respect to race and ethnicity in the work (e.g., the relevance of race and/or ethnicity to the study question, the rationale for use of a specific measure, the reason why a particular coding scheme was adopted, and the reason why an analytical approach or use of the variable was appropriate). Of the 329 studies which included data on participants’ race and/or ethnicity (Figure 1B), 29% provided a justification for at least 1 of their choices (data not shown). Without definitions and rationale from authors, it is unclear whether decisions to use an ethnoracial construct were intentional (e.g., motivated by theory or the study question) or unintentional (e.g., a limitation of the data structure, ritualistic or atheoretical practices). These choices can radically alter the construction of directed acyclic graphs or conceptual models, in addition to influencing subsequent analytical decisions. It is imperative that authors include definitions of race and ethnicity in their published work.

Highlighting this issue, we interpreted the use of “Caucasian” and “European American” to be signifiers of race (i.e., synonyms for “White”), though there is ambiguity in these terms. “European American” is murky: It can be interpreted as either a panethnic label (e.g., individuals of any race who identify as culturally German, Swedish, Romanian, etc.) or a racial signifier (e.g., a proxy for Whiteness). The term “Caucasian” arose in the 18th century as a “scientific” term for the “white race” (98). Advancements in genetics have debunked such attempts to construct biologically informed racial categories (99–104). Contemporarily, “Caucasian” refers to individuals from the Caucasus region, roughly spanning parts of Russia, Azerbaijan, Armenia, and Georgia (16, 98). Individuals from this region may not identify or be racialized as White. We collected data according to our a priori theoretical assumptions and knowledge of race and ethnicity in the United States. This may differ from the viewpoints or intentions of the authors of the sampled studies; however, without definitions and justifications, we were unable to assess intent.

Our findings highlight similar issues in the use of “Hispanic” and “Latino/a/x” in the absence of details on definitions, measurements, coding, and justifications. Some of the most common ethnoracial coding schemes were a variation of “White, Black, Hispanic, other.” This 4-level categorical coding scheme implies mutual exclusivity between groups. Our interpretation is that in such cases “Hispanic” is being treated as a de facto racial category, as the implied mutual exclusivity erases within-group racial heterogeneity and denies intersectionality. Such decisions have tangible consequences, including masking health disparities in Black Hispanic or Latino/a/x communities. This may further reinforce the racialized myth of mestizaje and that all Hispanic and Latino/a/x individuals occupy a “brown” or nonwhite racial identity (105). Again, without further information, it is unclear whether this coding scheme is the result of uncritical considerations of race—perhaps where groups are collapsed together in pursuit of a larger sample size—or represents a carefully considered ideological break from OMB definitions.

We acknowledge that ultimately what is communicated in published research is the result of tensions between the individual agency of the authors and meso-/macrolevel constraints by journals, funding agencies, and other institutions (106–108). Macrolevel structures that help shape the treatment of race and ethnicity include OMB and National Institutes of Health directives. In 1997, the OMB issued a government-wide standard for race and ethnicity data collection for the purposes of uniformity and comparability across studies that utilized federal data sets or were federally funded (106). The guidelines do not explicitly constrain researchers to only use particular measures of race and ethnicity, although in 2014 the OMB acknowledged that the “minimum reporting categories” have often been misinterpreted as the only permissible reporting categories and may have limited detailed racial and ethnic data collection and presentation (107, 109). Even if OMB guidelines are the limiting factor in how racial and ethnic data are collected or coded, we found that this motivation was only explicitly stated twice in our sample. Further, using guidelines set forth by the OMB does not negate the critical importance of communicating the measurement and coding of race and ethnicity data to ensure, as the OMB articulates, comparability across studies.

Recommendations provided by the journals themselves (mesolevel) may also influence this process. All 5 of the journals we studied have stated that they follow the International Committee of Medical Journal Editors’ guidelines, which since 2004 have included 2 specific recommendations for reporting on race and ethnicity (108, 110). The guidelines state that “authors should define how they determine race or ethnicity and justify their relevance” (108, p. 18). While guidance from journals may be in place, accountability for meeting said guidance is perhaps lacking.

Our work suggests that while epidemiology has made strides towards the greater inclusion of race and ethnicity data in mainstream research, there remains much to improve. Increased use of racial and ethnic data coupled with scant details as to measurement and coding may signal that critical consideration as to *why* race and ethnicity are important is missing. In essence, epidemiology may be perpetuating a continued practice of “ritualistic regression” (33) or “ritualistic inclusion” (29). As a discipline, we may recognize that race and ethnicity are important for understanding health stratification, but are unable to actualize that understanding into rigorous public health research and clear scientific communication.

We do not believe that the recommendations themselves need to be revisited (Table 1). The guidance provided in prior work is sound. Moreover, prior recommendations are not calling for radical change or for every researcher to become a race scholar. Rather, they call for adherence to core scientific principles: to motivate the inclusion or exclusion of specific data or persons in a study; to define constructs, especially those for which there is debate or ambiguity; to select construct measures best fit for a specific research question; to strike a balance between theoretical knowledge and practical constraints when coding variables; to engage with analytical methods appropriate for the study question; to interpret findings with care; to address the limitations of data, measures, coding, and methods; and to clearly communicate and justify all of the above in publication. What we need now is more adherence and accountability to the guidelines in order to push science forward. Practical thoughts on how to meet these recommendations have been offered elsewhere (111).

### Limitations

While we attempted to standardize data entry as much as possible and employed numerous data quality checks, data always retain a degree of subjectivity. An additional limitation is the measurement of ethnicity. While some scholarship has acknowledged the need for multidimensional ethnicity measurement (112), at the time of our study design, limited theory on the multiple measures of ethnicity for the United States had been proposed (113). Thus, we adapted Roth’s work (68). Future work should expand theorization on the breadth of ethnic and ethnoracial constructs.

Another limitation was the study time frame. We were originally motivated by LaVeist’s call to action (49). Given the time lag between article submission and publication, we believe articles published in 1995–1999 are still emblematic of the practices LaVeist originally critiqued. However, we recognize that articles published in 1990–1994 may provide even greater contrast.

Our study was also limited in its assessment of racial, ethnic, and ethnoracial coding schemes. As aforementioned, we collected either the analytical or descriptive codings, but not both. We frequently observed differential coding schemes between demographic and analyses tables in the same article. For example, investigators in some studies reported the proportion of the study population that self-classified as White, Black, Asian, Hispanic, or Native American/Alaska Native in their demographics table but then used the categorization “White, Hispanic, other” as an adjustment variable in analyses. There are probably differential practices and beliefs behind descriptive and analytical coding schemes, which we were unable to capture based on the design of our REDCap form.

### Conclusion

We echo LaVeist’s original imperative, as his words could not be truer today as the COVID-19 pandemic has reified chasmic health inequities within the United States (49, p. 26):

The question is not whether we should continue to conduct research on race, racism and health. The volume of research demonstrating race-associated differences in morbidity and mortality makes it clear that continued research is needed. And, as the health profile of America has been generally improving, the gap between black and white Americans persists. These well-established facts evince a need for continued research. But, it is not merely a matter of conducting more studies. What is *not* needed is more of the same.

When it comes to race and ethnicity in epidemiologic research, the recommendations are the same. It is simply time that we follow them.

## Supplementary Material

Web_Material_kwac146
