# Supplementary material for: Conceptualization, Operationalization, and Utilization of Race and Ethnicity in Major Epidemiology Journals, 1995–2018: A Systematic Review
Source: Am J Epidemiol. 2022 Aug 8;192(3):483–96. doi: 10.1093/aje/kwac146 (PMC9985111; doi:10.1093/aje/kwac146)
Supplement: Web_Material_kwac146 [file web_material_kwac146.zip › kwac146 Martinez Web material.pdf]

WEB MATERIAL

Conceptualization, Operationalization, and Utilization of Race and Ethnicity in Major Epidemiology Journals, 1995–2018: A Systematic Review

Rae Anne M. Martinez, Nafeesa Andrabi, Andrea N. Goodwin, Rachel E. Wilbur, Natalie R. Smith, and Paul N. Zivich

Table of Contents

Web Appendix 1 2

Web Appendix 2 3

Web Table 1 8

Web Table 2 9

Web Table 3 10

Web Table 4 14

Web Appendix 1

To reduce ineligible articles, the following search criteria were included:

```
(English[Language]) NOT (Letter[Publication Type]) NOT (Comment[Publication Type])
NOT (Editorial[Publication Type]) NOT (Review[Publication Type]) NOT
(News[Publication Type]) NOT (Case Reports[Publication Type]) AND (("United
States"[MeSH]) OR ("United States"[tw]) OR America[tw] OR "U.S."[tw] OR "US"[tw]).
```

Web Appendix 2

This form shows all questions. The electronic REDCap platform allows for skip patterns, which were used in this study. Skip patterns not shown.

Epid Article Abstraction

SAMPLE DATA EXTRACTION FORM. This form shows all questions. The electronic REDCap platform allows for skip patterns, which were used in this study. Skip patterns not shown.

Study ID	[Pre-populated field]

From PubMed Abstraction

Title	[Pre-populated field from PubMed]
First Author	[Pre-populated field from PubMed]
Publication Year	[Pre-populated field from PubMed]
PMID	[Pre-populated field from PubMed]
Journal	[Pre-populated field from PubMed]

Inclusion/Exclusion

Include?	◯ Yes<br>◯ No<br>(Must be: English language, U.S. Research, Human Subjects Must NOT be: Letter to the editor, Review/meta analysis, Mathematical, hypothetical, or simulation study. Include a study if it meets the above, but does not mention race.)
Reason for exclusion?	__________________
Study Design	◯ Randomized trials that include: RCT & cluster trials<br>◯ Cohort: long. obs. data & single time-point obs. data<br>◯ Case-control<br>◯ aggregate data (ecological level; no individual level outcomes)
Data Source	__________________<br>(Ex. Add Health, NHANES. Put NS if not stated. Do not use quotes or commas. Use semicolons to separate group, if multiple datasets were used.)
Sample size	__________________<br>(Put NS if not stated; Look first in the abstract, then methods, then table 1.)

Did they measure RACE?

◯ Yes
◯ No

Did they measure ETHNICITY?

◯ Yes
◯ No

Did they combine RACE and ETHNICITY?

◯ Yes
◯ No

What type of health outcome did they measure?

☐ Health behaviors (ex. Smoking, sexual behaviors, physical activity, diet/nutrition)
☐ Mental or physical health outcomes (ex. Obesity, high blood pressure, depression, cancer)
☐ Healthcare access or utilization (ex. Health insurance status, no. primary care visits, quality of life, quality of care)
☐ Other
(Select all that apply.)

What is the specific health outcome?

____

(Do not use quotes or commas. Use semicolons to separate multiple outcomes.)

Conceptualization of Race

Did the authors provide a working definition of race?

◯ Yes
◯ No
(Ex." Race is a social construct that..." "Race is a biological trait that...")

What definition was used?

____

(Do no use quotes. Provide page number of quote as (pg. XX).)

Conceptualization of Ethnicity

Did the authors provide a working definition of ethnicity?

◯ Yes
◯ No
(Ex." Ethnicity is a social construct that..." "Ethnicity is a biological trait that...")

What definition was used?

____

(Do no use quotes. Provide page number of quote as (pg. XX).)

Operationalization: Dimension of RACE - what question did they ask?

What dimension of RACE was used?

- ☐ Racial identity (subjective self-identification not set by pre-determined options)
- ☐ Racial self-classification (self-identification with PRE-DETERMINED options)
- ☐ Observed (by an interviewer)
- ☐ Reflected race (race you believe others assume you to be)
- ☐ Phenotype (Skin tone and other characteristics)
- ☐ Racial ancestry
- ☐ Not stated/Unclear/Other
- ☐ Not used
- ☐ Unclear between identity and self-classification

(Select all that apply (adapted from Roth 2016; see cheat sheet))

If unclear/other, add explanation

____

Operationalization: Coding of RACE - how were groups coded or collapsed?

What coding of RACE did they use?

____

(Do not use quotes or commas. Use semicolons to separate groups. Put NS if not stated.)

Operationalization: Dimension of ETHNICITY - what question did they ask?

What dimension of ETHNICITY was used?

- ☐ Ethnic identity (subjective self-identification not set by pre-determined options)
- ☐ Ethnic self-classification (self-identification with PRE-DETERMINED options)
- ☐ Country of Origin
- ☐ Ethnic ancestry
- ☐ Reflected ethnicity (ethnicity you believe others assume you to be)
- ☐ Observed ethnicity (by an interviewer)
- ☐ Not stated/Unclear/Other
- ☐ Not used
- ☐ Unclear between identity and self-classification

(Select all that apply (adapted from Roth 2016; see cheat sheet))

If unclear/other, add explanation

____

Operationalization: Coding of ETHNICITY - how were groups coded or collapsed?

What coding of ETHNICITY did they use?

____

(Do not use quotes or commas. Use semicolons to separate groups. Put NS if not stated.)

Analyses (Use)

What reference group was used?

____
(This is the racial or ethnic group that is used as the referent. Do not use quotes or commas. Use semicolons to separate groups. Put NS if not stated.)

Predictive model?

- ◯ Yes
- ◯ No

(Model to be used for prediction, rather than for hypothesis testing or causal inference.)

How was race and/or ethnicity used?

- ◯ OF INTEREST: focal IV, modification analyses, mediation analyses
- ◯ NOT OF INTEREST: control, covariate, confounder, matching criteria
- ◯ EXCLUSION CRITERIA: to exclude people from analyses
- ◯ OTHER

If other use, add explanation

____

Justifications of Race

Did the authors provide a justification for any of their choices regarding race?

- ◯ Yes
- ◯ No

(Justifications include but is not limited to: (1) why race was important to the study or why racial information was collected, (2) why race was measured as a specific dimension, and (3) why racial groups were collapsed in coding. Please be a broad as possible.)

Please copy-paste all justification language from the text here.

____
(Do no use quotes. Provide page number of quote as (pg. XX).)

Justifications of Ethnicity

Did the authors provide a justification for any of their choices regarding ethnicity?

- ◯ Yes
- ◯ No

(Justifications include but is not limited to: (1) why ethnicity was important to the study or why ethnic information was collected, (2) why ethnicity was measured as a specific dimension, and (3) why ethnic groups were collapsed in coding. Please be a broad as possible.)

Please copy-paste all justification language from the text here.

____
(Do no use quotes. Provide page number of quote as (pg. XX).)

**Other**	
Flag this article as a "special case" (i.e., requires whole group discussion)	◯ Yes<br>◯ No
Flag this article for "in-text" discussion (i.e., potentially warrants mention in the manuscript discussion section)	◯ Yes<br>◯ No
Open-ended notes about important / noteworthy things	__________________________________<br>(Please enter initials & date reviewed here (ex. RAM 2020/03/31))

Web Table 1. Characteristics of studies without race data (*n* = 101)

Study Characteristic	1995–99 (*n* = 23)		2000–04 (*n* = 30)		2005–09 (*n* = 19)		2010–14 (*n* = 15)		2015–18 (*n* = 14)	
	No.	%	No.	%	No.	%	No.	%	No.	%
Study design										
RCT	0	0	0	0	0	0	0	0	0	0
Cohort	21	91	26	87	15	79	14	93	13	93
Case-control	2	9	4	13	4	21	1	7	1	7
Ecological	0	0	0	0	0	0	0	0	0	0
Study outcome[a]										
Health behavior	2	9	1	3	1	5	0	0	3	21
Physical or mental	15	65	23	77	15	79	12	80	8	57
Health-care access	1	4	1	3	0	0	0	0	0	0
Other	6	26	6	20	3	16	3	20	4	29
Sample size, no.[b]										
<1,000	8	32	14	39	5	25	7	44	5	36
1,000-5,000	9	36	8	22	6	30	1	7	2	14
5,001–10,000	2	8	2	6	1	5	3	19	0	0
10,001–100,000	5	20	11	31	4	20	2	13	2	14
>100,000	0	0	1	3	3	15	3	19	3	21
Missing	1	4	0	0	1	5	0	0	2	14

RCT, randomized controlled trial.

[a] Study outcomes were classified as health behaviors (e.g., smoking, dietary intake, physical activity, sexual behaviors), mental or physical health (e.g., obesity, high blood pressure, cancer, depression), health care access of utilization (e.g., health insurance status, number of primary care visits, quality of care), or other. Study outcomes are not mutually exclusive and may sum to more than 100%.

[b] Some studies listed more than one analytical sample size; values may sum to more than 100%.

Web Table 2. Racial coding schemes (*n* = 138)

Coding Scheme[a]	No.	%
Black, White	29	21
Black, other, White	18	13
NS (not stated)	17	12
White	16	12
Non-White, White	15	11
Black, non-Black	5	4
Black	4	3
Caucasian	4	3
Black, other, unknown, White	3	2
Other, White	3	2
White/Caucasian	2	1
Asian, Black, other, unknown/missing data, White	1	1
Asian, Black, White	1	1
Asian/Pacific Islander, Black, Native American, other/unknown, White	1	1
Black, Caucasian, other	1	1
Black, missing, other, White	1	1
Black, non-Hispanic White	1	1
Black, other than Black and White, White	1	1
Black, unknown, White	1	1
Caucasian only, non-Caucasian	1	1
Caucasian, non-White	1	1
Caucasian, other	1	1
European American	1	1
Missing, other/multiple, White	1	1
Non-Hispanic Black	1	1
Non-Hispanic Caucasian	1	1
Non-Hispanic White	1	1
Non-Hispanic White, other	1	1
Non-White, White/Caucasian	1	1
Not White, White	1	1
Other race, White	1	1
Other/unknown, White	1	1
White race/ethnicity	1	1

[a] Across stratum, 33 unique racial coding schemes were identified from among 138 coding schemes belonging to 137 studies. Number of coding schemes exceeds number of studies, as collection of multiple analytical coding schemes was allowed. These studies included racial data and may have included ethnicity data, but did not combine the two into an ethno-racial construct. Information on capitalization was not collected. No attempt was made to collapse coding schemes based on similarity.

Web Table 3. Ethnoracial coding schemes (*n* = 180)

Coding Scheme[a]	No.	%
African American, White	10	6
African American, other, White	7	4
Asian, Black, Hispanic, other, White	7	4
Black, Hispanic, White	7	4
Black, Hispanic, other, White	6	3
Hispanic, non-Hispanic Black, non-Hispanic White, other	6	3
African American	5	3
Non-Hispanic Black, non-Hispanic White	3	2
Non-White, White[b]	3	2
African American, European American	2	1
Black, other, White[b]	2	1
Black, White[b]	2	1
Black/African American	2	1
Foreign-born Hispanic, non-Hispanic White, other, US-born Hispanic	2	1
Mexican American, non-Hispanic Black, non-Hispanic White, other	2	1
African American, Asian, Hispanic, Native American, other, refused/unknown, White	1	1
African American maternal race	1	1
African American or Black	1	1
African American race	1	1
African American, Anglo-American, Latino	1	1
African American, Asian American, Caucasian, other	1	1
African American, Asian, Caucasian, Hispanic, other	1	1
African American, Asian, Caucasian, other	1	1
African American, Asian, Hispanic, Native American, other, White	1	1
African American, Asian, Hispanic, Native American, other, White	1	1
African American, Asian, Hispanic, other, White non-Hispanic	1	1
African American, Asian, Latina, partly Native American, White	1	1
African American, Asian, Latino, non-Hispanic White, other	1	1
African American, Asian, Latino, other, White	1	1
African American, Asian/other, Latino, White	1	1
African American, Caucasian	1	1
African American, Caucasian, Japanese American, Latino, Native Hawaiian	1	1
African American, Caucasian, not stated, other	1	1
African American, Caucasian, other	1	1
African American, Caucasian/other	1	1
African American, Chinese American, Hispanic, White	1	1
African American, Chinese, Hispanic, Japanese, White	1	1
African American, Cuban American, non-Hispanic White	1	1

African American, Hispanic, non-Hispanic White	1	1
African American, Hispanic, non-Hispanic, White/Caucasian	1	1
African American, Hispanic, other, White	1	1
African American, Hispanic, White	1	1
African-American, Hispanic, White	1	1
African American, Hispanic, White/non-Hispanic	1	1
African American, Hispanic/other, White	1	1
African American, Latino, other, White	1	1
African American, Latino, White	1	1
African American, Mexican American, non-Hispanic White, Puerto Rican	1	1
African American, mixed race/ethnicity, other, White	1	1
African American, non-African American	1	1
African American, other	1	1
African American, other race, White	1	1
African American, other race/ethnicity	1	1
African American/Black, White	1	1
African-American non-Hispanic, Asian, Dominican, Mexican, other, other Hispanic, Puerto Rican, White non-Hispanic	1	1
African-American race, non-African-American race	1	1
African-American, Asian-American, Latina, White	1	1
African-American, Asian, Caucasian, other unknown	1	1
African-American, Asian/Pacific Islander, Hispanic, non-Hispanic White	1	1
Alaska Native, American Indian, Asian or Pacific Islander, biracial, Hispanic or Latino or Latina, minority (African American or Black), multiracial, Native Hawaiian, non-minority (non-Hispanic White), other	1	1
Alaska Native/American Indian/Native Hawaiian or Pacific Islander or multiracial, Black, Hispanic, White	1	1
All other races and ethnicities, non-Hispanic White	1	1
All others/White and others, Black	1	1
American Indian[b]	1	1
American Indian, Asian or Pacific Islander, Hispanic or Latino, non-Hispanic Black, non-Hispanic White, other non-Hispanic (including multiracial)	1	1
American Indian, Asian, Black, Hawaiian, unknown, White	1	1
American Indian, Asian, Black, Hispanic, other, unknown, White	1	1
American Indian, Asian/Pacific Islander, Black, Hispanic, other/unspecified, White	1	1
American Indian, Asian/Pacific Islander, Black, Hispanic, unknown, White	1	1
Asian American, Black, Latino, Native American, White	1	1
Asian and Pacific Islander, Black non-Hispanic, Hispanic, other, White non-Hispanic	1	1
Asian or Pacific Islander, Hispanic, non-Hispanic Black, non-Hispanic White	1	1
Asian, Black, Hispanic, non-Hispanic White	1	1

Asian, Black, Hispanic, White	1	1
Asian, Hispanic, non-Hispanic Black, non-Hispanic White, other race or multiracial	1	1
Asian, Hispanic, non-Hispanic Black, non-Hispanic White, other/unknown	1	1
Asian, Hispanic, non-Hispanic Black, non-Hispanic White, Pacific Islander or American Indian	1	1
Asian, Hispanic, non-Hispanic Black/African American, non-Hispanic White, other	1	1
Asian/Pacific Islander, Hispanic, non-Hispanic Black, non-Hispanic White, other	1	1
Asian/Pacific Islander, Black, Hispanic, Native American, White	1	1
Black non-Hispanic, Hispanic (any race), other, White non-Hispanic	1	1
Black non-Hispanic, Hispanic, other non-Hispanic, White non-Hispanic	1	1
Black non-Hispanic, Hispanic, White non-Hispanic	1	1
Black or African American, Chinese American, Hispanic, White or Caucasian	1	1
Black, Caucasian, Hispanic, other	1	1
Black, foreign-born Latina, other, US-born Latina, White	1	1
Black, Hispanic, other	1	1
Black, Hispanic/Latino ethnicity, White or other	1	1
Black, Hispanic/Latino, other, White	1	1
Black, Mexican, other, White	1	1
Black, non-Black[b]	1	1
Black, other (Hispanic, Asian, other, not identified), White	1	1
Black/African-American, White	1	1
Caucasian, Chinese, Filipino, Hawaiian/part-Hawaiian, Japanse	1	1
Caucasian, Hispanic, non-White	1	1
Caucasian, Japanese American, Native Hawaiian	1	1
Chinese American, Hispanic, Japanese American, non-Hispanic Black, non-Hispanic White	1	1
Chinese, Filipina, other Asian Americans, Native Hawaiians and Pacific Islanders (AANHPI)	1	1
Chinese, Filipino, Korean, Latino, non-Hispanic White, other Asian, Vietnamese	1	1
Chinese, Filipino, multiracial/ethnic, other Asian alone, Vietnamese	1	1
Chinese, Hispanic, non-Hispanic Black, non-Hispanic White	1	1
Hispanic Mexico-born, Hispanic US-born, non-Hispanic Black, non-Hispanic White	1	1
Hispanic, African-American/non-Hispanic Black, non-Hispanic Asian, non-Hispanic White, other	1	1
Hispanic, Mexican American, non-Hispanic Black, non-Hispanic White, other	1	1
Hispanic, missing, non-Hispanic Black, non-Hispanic White, other	1	1
Hispanic, non-Hispanic African American, non-Hispanic White, other	1	1

Hispanic, non-Hispanic Asian, non-Hispanic Black, non-Hispanic White, other non-Hispanic (including American Indian, Hawaiian, and other Pacific Islander)	1	1
Hispanic, non-Hispanic Asian/Pacific Islander, non-Hispanic Black, non-Hispanic White	1	1
Hispanic, non-Hispanic Black, non-Hispanic multiracial, non-Hispanic other, non-Hispanic White	1	1
Hispanic, non-Hispanic Black, non-Hispanic other, non-Hispanic White	1	1
Hispanic, non-Hispanic Black, non-Hispanic White	1	1
Hispanic, non-Hispanic White	1	1
Hispanic, White	1	1
Hispanic/Latino, non-Hispanic Black, non-Hispanic White, other race/ missing (including Asians)	1	1
Hispanic/mixed race/other, non-Hispanic Black, non-Hispanic White	1	1
Latina, non-Latina Black, non-Latina White, other/missing	1	1
Mediterranean, non-White, other ancestry, other Caucasian, Scandinavian	1	1
Minority race/ethnicity	1	1
Missing, non-Hispanic White, other	1	1
Non-Hispanic Black, non-Hispanic White, other	1	1
Non-Hispanic Black, non-Hispanic White, other, unknown	1	1
Non-White/Hispanic, White non-Hispanic	1	1
Non-White (Black, Latina, Asian), White	1	1
Non-White or Hispanic, White	1	1
Non-White, White non-Hispanic	1	1
Other, White non-Hispanic	1	1
White[b]	1	1
White (including Mexican, Puerto Rican, and all other Caucasian)	1	1
White, Black, Hispanic, Asian/Pacific Islander, other/unknown	1	1

[a] Across stratum, 129 unique racial coding schemes were identified from among 180 total coding schemes belonging to 176 studies. These 176 studies combined the racial and ethnic data into an ethno-racial construct or operationalized an ethno-racial construct. The number of coding schemes exceeds the number of studies, as collection of multiple analytical coding schemes was allowed.
[b] These coding schemes appear identical to a few of the racial coding schemes (Web Table 2); studies associated with these coding schemes did use an ethno-racial construct but the variable recoding obscured this. For example, a study may treat "Hispanic" or "Latino" as a racial category and then recode to a binary variable of "non-White, White," where "non-White" includes "Hispanic, Black, Asian, and Native American/Alaskan Native" individuals. Information on capitalization was not collected. No attempt was made to collapse coding schemes based on similarity.

Web Table 4. Role of race and/or ethnicity in analyses, 1995–2018

Analytical Role[a]	1995–99 (*n* = 73)		2000–04 (*n* = 67)		2005–09 (*n* = 73)		2010–14 (*n* = 55)		2015–18 (*n* = 61)	
	No.	%	No.	%	No.	%	No.	%	No.	%
"Of interest"	23	32	13	19	25	34	12	22	19	31
"Not of interest"	33	45	44	66	41	56	36	65	39	64
"Exclusion"	15	21	9	13	7	10	7	13	2	3
"Other"	2	3	1	1	0	0	0	0	1	2

[a] "No. Included" indicates the number of articles which included data on race and/or ethnicity.
